# Benedictine

**Published:** 1857-08

**Authors:** 


### BENEDICTINE.

WE propose to "Lord" Alexander, of Kentucky, the Lenox, the Stuart, the Douglass, and other millionaire bachelors, to Howardize themselves, by founding an institution for qualifying American born girls to be cooks, nurses and housemaids, and thus, in some slight measure, atone for their celibate dereliction, and the wives of New York will rear a monument to their memories, more immortal than the pyramids

